# Supplementary material for: Are carbon emissions trading and green financial instruments synergistic? -Comprehensive quantitative research based on content analysis
Source: PLoS One. 2024 Mar 7;19(3):e0298601. doi: 10.1371/journal.pone.0298601 (PMC10919601; doi:10.1371/journal.pone.0298601)
Supplement: S1 Data — (ZIP) [file pone.0298601.s001.zip › Data/地级市Z_F工作报告.zip!/╣π╬≈/┴°╓▌╩╨/└┤╫╘╣1⁄2╓┌║┼í╛┬φ┐╦╩2╛▌═°í┐_2008.docx]

各位代表：

现在，我代表市人民政府向大会报告工作，请予审议，并请各位政协委员和列席人员提出意见。

2007年工作回顾

2007年，在市委的正确领导下，我们坚持以科学发展观统领全局，团结和依靠全市各族人民，朝着全面建设小康社会的奋斗目标又迈出了坚实的一步。全市工业化和城镇化进程进一步加快，“1123”工作目标全面完成，经济总量再上新台阶，各项经济指标创历史最好水平，为民办20件实事基本完成，经济社会实现了又好又快发展。过去一年，是全市经济快速发展的一年，是工业生产和效益双丰收的一年，是城市市容市貌和人居环境不断改善的一年，是文化繁荣、社会和谐稳定的一年，是柳州人民生活水平与经济发展水平同步提高的一年。

据初步统计，全市实现地区生产总值738.8亿元，增长16.5%，增幅为十二年来最高；财政收入首次突破百亿大关，达116.4亿元，增长22.3%；一般预算支出75.0亿元，增长19.8%；工业总产值1389.1亿元，增长24.8%；农林牧渔业总产值134.5亿元，增长7.3%；社会消费品零售总额274.1亿元，增长18.9%；全社会固定资产投资305.5亿元，增长52.2%。单位生产总值能耗下降4.5%，主要污染物排放总量减少10%以上。城镇居民人均可支配收入12866元，增长16.9%；农民人均纯收入第一次超过3000元，达3390元，增长16.4%。城镇登记失业率控制在4.33%以内，人口自然增长率控制在自治区下达指标以内。

一、工业经济总量大幅提升，工业发展迎来了改革开放以来的又一个最好发展时期

市委、市政府加强了对工业经济的领导，加大了对工业发展的扶持力度。制定《关于进一步加快推进新型工业化进程，建设全区率先实现工业化的示范城市的决定》，推出了促进工业发展、鼓励外贸出口、支持企业自主创新、鼓励企业搬迁改造和加强广告宣传等政策措施，适时调整“十一五”工业发展规划，将2010年的工业总产值目标由1600亿元调整为2400亿元。召开全市工业大会，对工业经济发展进行再动员再部署，组织开展“中小企业服务月”等活动，极力营造全社会关心支持工业发展的浓厚氛围。进一步完善了中小企业服务体系，市财政专门追加3000万元用于扶持中小企业发展；为市中小企业担保公司新注入资本金2.16亿元，年内担保业务总量突破10亿元，促进了大中型企业和中小企业共同发展。

实施四大成长计划成效显著，工业发展实现“三个突破”、“四个一百”。支柱产业发展取得“三个突破”：全市汽车产量突破60万辆，钢材产量突破600万吨，工程机械产量突破3万台。工业发展实现“四个一百”：全市工业投资突破100亿元，产值过亿元企业突破100家，柳工主营业务收入突破100亿元，柳江县工业产值突破100亿元。带动全市工业总产值净增300多亿元，约为全区工业增量的三分之一，全市工业总量三年内翻了一番。我市被确定为国家级汽车及零部件出口基地和汽车零部件生产基地。柳工装载机、网山白砂糖被评为2007年中国名牌产品；金嗓子喉宝润喉片等8个产品新获国家免检产品。园区经济快速发展，重点监测的六大工业园区完成工业总产值178.2亿元，增长77.4%。

工业投资再创新高，技改竣工项目为历年之最。全市完成工业投资116.1亿元，增长44.0%。竣工投产3000万元以上项目35项，上汽通用五菱新型发动机、东风柳汽重卡驾驶室、小型乘用车生产线等一批重点项目相继投产，柳工万台叉车、柳钢冷轧板带等一批重大项目开工建设，为“十一五”后三年持续快速发展打下了坚实基础。

工业经济效益大幅提升，工业发展进入良性循环轨道。全市规模以上工业产值和主营业务收入双双突破千亿大关，分别达1148.8亿元和1146.6亿元，增长26.5%和28.6%；实现利税总额139.8亿元，增长33.8%；其中利润总额70.9亿元，增长54.2%。工业发展呈现出收入增速高于产值增速、利税增速高于收入增速、利润增速高于利税增速的良好态势。

节能减排卓有成效，工业经济与环境保护同步推进。开展节能减排“百日行动”。全年各行业节能、环保投入达12亿元，创历史新高。全市规模以上工业万元增加值能耗降至2.63吨标准煤，下降13.95%。二氧化硫（SO2）减排1.36万吨，下降13.52%；化学需氧量（COD）减排1.83万吨，下降10.13%。我市全面完成了上级下达的节能减排任务，是全区排放总量下降最多的城市。循环经济取得新成效，全市有16个企业20多种产品通过资源综合利用产品认证，制糖企业基本实现资源综合利用，柳钢、柳化、鱼峰水泥等企业在废水循环、余热发电、废渣利用等方面取得了较好成绩。

二、社会主义新农村建设扎实推进，农村面貌出现新的改观

农业产业结构进一步优化。粮食生产稳中有升，全年粮食总产86.6万吨，增产1.0%。种蔗及养蚕成为农民增收的主要途径。糖料蔗种植面积145万亩，增长6.3%；糖蔗产量748万吨，增长13.7%，农民种蔗人均增收150元。桑蚕种养大幅增加，全市桑园面积达42.1万亩，增长36.5%；农民售茧收入6.98亿元，名列全区第二，增长26%，带动全市农民人均增收51.6元。养殖业稳步发展，全年肉类总产量26.6万吨，增长0.9%。实施农业产业化重点龙头企业成长计划，新增市级龙头企业15家。实施“千村两机”工程成效明显，农业机械化进程加快。

农村基础设施建设和社会主义新农村建设试点取得新进展。完成农村公路建设491.7公里，新增4个乡通油路，51个行政村通等级路，12个村通水泥路，166个自然村通屯级路。水利建设完成15座水库除险加固、11座水库穿坝涵管改建；建成沼气池1万座，改厕1.2万座，解决了5万农村人口饮水安全问题，圆满完成了全年任务。社会主义新农村建设试点由上年16个增至27个，三江程阳八寨等两个市级试点建设全年完成投入3358万元，自治区对我市新农村建设试点给予了充分肯定。

县域经济增长强劲。六县实现地区生产总值243.4亿元，增长25.4%；财政收入17.4亿元，增长22.6%；规模以上工业总产值158.4亿元，增长43.3%；全社会固定资产投资105.7亿元，增长53.1%。柳江再次评为广西经济发展十佳县，柳江、鹿寨跻身中国西部百强县。

扶贫开发取得新成果。农村劳动力转移和农村社会保障体系的有序推进，为农民脱贫致富打下了坚实基础。首批127个贫困村“整村推进”扶贫开发顺利通过自治区验收，第二批85个贫困村“整村推进”扶贫开发正式启动。市直机关、大中型企业与乡镇、村屯结对子帮扶活动取得较好成效。全年解决了2万贫困人口的脱贫问题。

三、服务业健康稳定发展，城市服务功能加快提升

市场建设和培育取得新进展，流通新业态发展迅速。城乡商品市场活跃，购销两旺，全年实现社会消费品零售总额274.1亿元，增长18.9%，是近十年来增长最快的一年。农贸市场改造、提升加快推进，广雅市场完成拆迁，东环市场主体工程完工，八桂农网和柳邕市场完成整合。“万村千乡市场工程”扎实推进，新建农家店470家，已覆盖全市100%的乡镇和70%的行政村。现代物流、电子商务发展迅速。桂中海迅河西工业物流配送中心一期工程建设完成，广西汽车贸易园投入运营，中铁柳州物流园正式开园。

市场调控力度加大，市场供需保持平衡。针对肉品市场供应紧张和肉价上涨形势，及时出台城市低保对象副食品价格补贴、企业生猪采购补贴、能繁母猪补贴等政策，积极组织生猪和肉源的采购及调运，确保了肉品供应和肉价稳定。加强市场监管，强化屠宰管理，以放心肉为重点的食品安全工程取得新的成效。农产品质量安全不断提高，新增无公害认证产品21个、绿色食品认证产品14个。

旅游业快速发展。三江、融水、融安旅游资源整合及“百里柳江”、“百里风情”等重大旅游项目建设加快推进，程阳八寨入选首批“中国景观村落”，以蟠龙山景观瀑布为代表的柳江夜景成为城市旅游的新亮点。成功举办“国际小丑艺术节”，假日旅游取得较好成效。全年接待国内外游客790万人次，增长15%；接待入境过夜旅游者5万人次，增长26.1%。

四、城市规划建设管理成效显著，山清水秀地干净的城市新风貌日益凸显

城市规划和土地管理得到加强。城市总体规划、土地利用总体规划修编工作加快推进，“百里柳江”、河东中心区、柳东新区规划不断完善，规划水平进一步提高。开展查处违法占地违法建设“百日行动”，切实保护耕地；推进土地资源的整合利用，提高了土地利用效率。

城市基础设施建设加快推进。全市基本建设投资完成95.7亿元，增长40.9%；其中重点工程项目完成投资56亿元，创历史新高。城市路网进一步完善。柳太公路、阳和南路基本完工，南环路西段、东堤路（二期）抓紧续建，雅儒路、柳长路、迎宾路等道路改造建设按期开工。柳东新区基础设施建设加快。静兰桥改建顺利推进，火炬大道开工建设，柳东新区及河东片区路网、管网及供电设施不断完善。旧城改造成效明显。穿衣戴帽工程和城中村改造加快实施，三中路、弯塘路示范街及小街小巷改造完成，跃进路、北站路温馨工程建设加快。夜景灯光工程全面实施，龙城夜色更具神韵。城市防灾系统进一步完善，防洪工程木材厂堤建设完成。房地产业健康发展，全年完成投资78.7亿元，增长74.1%

“碧水蓝天、青山绿地”工程深入实施。柳钢、柳化、柳州有色冶炼、柳兴制糖等企业污染治理取得新进展。环保基础设施建设加快。白沙污水处理厂、龙泉山污水处理厂（二期）建设基本完成，竹鹅溪主河道治理改造基本完工，城市公厕和垃圾转运站（一期）加紧建设。园林景观建设不断加强。河西花卉公园（一期）、蜡烛山公园（一期）、羊角山公园、河东公园及城市绿地系统（一期）基本建成，全民义务植树造林活动全面开展，完成了10条小街小巷“一巷三分绿”试点，新增城市绿地271.9公顷。柳江河饮用水保护河段保持国家地表水Ⅲ类水质标准，在全区37条河流93个重点河段的水体水质评价中位居第一，自治区为此在我市举办了广西环保人柳江漂游活动。市区环境空气质量全年优良天数达351天，为20年来最好。

城市管理进一步加强。“城乡清洁工程”深入实施，城市保洁标准提高，对重点路段、重点部位及农贸市场、城乡结合部的市容环境治理取得成效。城市管理综合执法全面实施，“数字城管”①建设开始启动。出台实施城市养犬管理暂行办法。免费开放市属公园和环卫公厕，深受市民群众欢迎。

五、民生不断改善，社会保持和谐稳定

就业再就业工作取得新成绩。全面落实新一轮就业再就业扶持政策，初步建立了就业困难人员动态援助长效机制。全市城镇新增就业人数4.8万人，其中下岗再就业人员1.68万人，大龄就业困难对象再就业6515人，累计消除零就业家庭1458户。统筹推进城乡就业，全市农村新增转移就业5.6万人，转移就业职业培训4.8万人。

社会保障体系进一步完善。加强社会保险扩面征缴，基金征缴率保持较高水平。全市企业退休人员月均养老金由800元提高到894元，城镇居民最低生活保障标准由210元提高到240元，社区、村委会工作人员生活补贴标准也得到相应提高。建立了农村最低生活保障制度，落实农村五保供养制度；启动城镇居民基本医疗保险国家级试点，全面铺开城乡医疗救助，制定出台农民工参加医疗保险和工伤保险政策。深入实施扶助残疾人的“阳光计划”，改造农村残疾人危房1035户，超额完成年度任务。农村特困户茅草房改造完成1726户。救灾求援机制不断完善，春荒、夏涝和村寨火灾的受灾群众得到妥善安置。实施困难家庭安居工程，全年新开工建设经济适用房和廉租房3000多套、32.5万平方米，符合条件的困难家庭全部享受到了廉租房政策。

社会大局保持稳定。深入推进平安柳州建设，积极开展“两抢一盗”、“夏季风暴”等专项整治行动和“三大纠纷”调处加强年专项活动，加大对六合彩、非法传销、“黑网吧”、“黄赌毒”的打击查处力度，社会治安状况进一步好转，全市刑事案件下降2.9%，命案破案率达93.9%。加强信访工作，进一步畅通了信访渠道。安全生产得到加强，我市连续七年没有发生一次死亡10人以上的重大安全生产事故，安全生产工作全区领先。

六、各项改革不断深化，对外开放出现新的局面

国有企业、投融资体制等改革顺利推进。市属国有企业的公司化改造基本完成。柳化集团改制重组顺利推进，工贸股份和百货股份整合改制正在实施。中糖股份和柳地建总在上海产权交易所挂牌交易。金融服务体系改革步伐加快，融资工作取得新突破，柳钢股份、优联股份成功上市，城投公司成功发行10亿元债券。国有资产经营公司资源整合有效推进，产业、基础建设与公共事业、金融与担保三类新型投融资平台初步建成。金融对地方建设的支持力度加大，金融机构年末存款余额725.3亿元，增长13.7%；贷款余额461.7亿元，增长16.1%；存贷比为63.65，比上年上升1.4个百分点。

招商引资成绩显著。不断创新招商方式方法，先后在广州、深圳、香港等地开展招商推介活动，积极参加第四届中国-东盟博览会，招商引资再结硕果。全年市外境内资金实际到位156.1亿元，增长53.8%。实际利用外资8239万美元，增长26.4%。新批外商投资企业25家，增资12家。工业利用外资保持良好势头，五菱集团与香港俊山集团成立合资公司，柳化正在引进战略投资筹建大型化工集团。承接东部产业转移取得新进展，全市园区承接产业转移企业43家。

外贸出口再创新高。全市实现进出口总值12.8亿美元，增长18.5%，其中出口6.7亿美元，增长46.2%。出口产品结构继续改善，机电和钢铁产品出口大幅增长，柳钢、柳工分列全区出口50强企业第一、第二位。

七、各项社会事业全面发展，精神文明建设进一步加强

科技创新全面推进。第三轮创新计划全面完成，有12项成果获广西科技进步奖，柳工《机电一体化、高原型特种轮式装载机》获特别贡献奖。重点科技项目实施效果明显，有两个项目列入“国家科技支撑计划”。自主创新能力增强，53%的规模以上工业企业开展了信息化建设，49家企业被认定为自治区级制造业信息化应用示范企业，新增自治区级工程技术研究中心2个，东风柳汽推出“风行景逸”首款自主品牌家用两厢车。人才工作得到加强，新增自治区人才小高地2个。成立留学人员创业园，全年引进企业7家。

教育事业蓬勃发展。基础教育、农村教育不断加强，我市为自治区通过国家“两基”攻坚评估验收做出突出贡献，被自治区评为“两基”攻坚先进单位；优质普通高中教育资源得到拓展，新增两所自治区示范高中。教育教学质量稳步提高，我市高考成绩继续名列全区前茅。职业教育快速发展，完成了柳州运输职业技术学院、城市职业学院的资源整合，柳州职业技术学院列入国家示范性高等职业院校建设计划。教育扶贫行动力度加大。“两免一补”政策全面落实，贫困住宿生生活补助标准由上年每生每年100元提高到160元。共资助1000名贫困学生和62名贫困残疾学生免费就读中等职业学校，免除2608名城市“低保”家庭子女就读中小学和职校的学杂费。建立资助贫困家庭子女上大学工作机制，共资助2268名贫困大学新生入学。着力解决农民工子女就学问题，新增农民工子女定点就读小学6所、中学2所，在校生均享受同等待遇。

文化体育事业加快发展。《八桂大歌》实现全国商业化巡演。柳州博物馆新馆投入使用。柳州山歌等6个项目列入第一批自治区级非物质文化遗产名录。基层公共文化服务体系不断完善，社会群众文化活动广泛开展。软件正版化工作顺利开展。“扫黄、打非”及对网吧、娱乐场所和出版物市场的专项整治得到加强。边远山区广播电视收视收听条件进一步改善。实施“农家书屋”工程建设，建成农家书屋150家。成功举办首届“城市之光”运动会，全区第十一届运动会我市奖牌总数和团体总分位居第二，成功承办7项国际国内赛事并获得多方好评。

卫生和人口计生工作得到加强。构建城市社区卫生服务体系，已建社区卫生服务机构101个，完成规划的75%。市人民医院新院、市妇幼保健院大楼即将投入使用。新型农村合作医疗全面推进，全市参合率达79.62%，受益群众超过50万人。农村卫生基础建设持续加强，200个行政村卫生所全部竣工；国债资金乡镇卫生院项目正在抓紧建设。爱国卫生运动深入开展，重大传染病预防控制卓有成效。人口和计划生育工作新机制初步建立，计划生育率为81.56%。

精神文明建设活动深入开展。以社会主义荣辱观为主题的公民道德教育扎实推进。未成年人思想道德教育得到加强。妇女儿童发展纲要全面落实。民族工作继续加强，少数民族贫困地区发展加快，各民族团结和睦。国防建设、双拥共建不断加强，我市连续第六次获“全国双拥模范城”称号。

八、作风效能建设成效显著，政府工作不断改进

作风效能建设取得阶段性成果。全面落实首问负责制、限时办结制、责任追究制，率先在全区推行否定报备制。行政审批制度改革不断深化，92%的行政审批事项办结时限缩减50%以上。改造升级电子政务系统，实现了所有党政机关、事业单位和大部分大中型企业的电子公文交换。建立健全市、县（区）两级政务服务中心，构建三级联网电子监察系统，实现了对重点单位重点事项的实时监控。打造“12345”政府服务平台，群众投诉转办反馈率由60%提高到90%。加强政风建设，行政机关向社会公开承诺，接受市民监督和评议。联动开展民主评议行风政风、政务公开和效能监察工作并取得良好成效。自治区对我市作风效能建设给予充分肯定，先后两次在我市召开作风效能建设现场会。

民主法治和党风廉政建设得到加强。市政府自觉接受市人大、市政协的监督，广泛听取社会各方面的意见和建议，认真办理人大代表议案、建议和政协委员提案，全年办理市人大代表议案建议135件，政协提案200件，办结政协建议案2件，答复率均为100%。加强司法基层建设，深入推进依法治市工作。法律援助工作不断规范，全年援助案件增长180%，受援人数超过万人。创新行政复议和政府法制监督工作方式，推行行政复议庭审听证制度。加强政府投资审计监督，资金使用效益进一步提高。全面落实党风廉政建设责任制，坚持纠建并举推进政风行风建设，政务环境进一步优化。

一年来，我市人事、编制、外事、侨务、口岸、统计、物价、审计、档案、人防、气象、粮食、农机、宗教、老龄、修志、防震减灾、社会科学、机关事务管理等部门和单位都做了大量的工作，取得了新的成绩。金融、工商、税务、质监、药监、电力、电信、邮政、铁路、民航、海关、海事、边防、烟草、检验检疫、国家安全等中央自治区驻柳单位为我市经济社会发展提供了有力保障，做出了积极贡献。

总结一年来的工作实践，我们深刻体会到，要在新时期实现经济社会的又好又快发展：一是必须坚持以科学发展观统领全局，牢牢把握加快发展第一要务，抢抓机遇促发展，不断提高应对困难和挑战、更好地在国家宏观调控下创造性地加快发展的能力；二是必须坚定不移地走有柳州特色的新型工业化道路，坚持用工业的理念谋划农业发展，坚持依托一产、二产加快服务业发展，坚持经济发展与宜居城市建设同步推进，全面加快工业化和城镇化进程；三是必须坚持以人为本，关注民生，扎扎实实为民办实事，不断加强经济社会发展的薄弱环节，促进社会和谐稳定，让人民群众充分享受到改革发展的成果；四是必须坚决革除阻碍发展的一切体制性障碍，坚持走开放合作、互利共赢的发展道路，大力发展外向型经济，进一步确立柳州在区域经济中的地位和优势；五是必须充分调动各方面的积极性，凝聚社会各界力量，营造全市上下齐心协力、干事创业的良好氛围，形成推动发展的强大合力。这些宝贵的经验，对我市今后的发展将会起到积极的推动作用。

各位代表，2007年是我市经济社会大步向前发展的一年。这是市委正确领导及市人大、市政协监督支持的结果，是全市人民团结奋斗和社会各界大力支持的结果。在此，我代表市人民政府，向全市人民，向驻柳人民解放军指战员、武警部队官兵、政法干警，向各民主党派、工商联、人民团体及海内外所有关心和帮助我市建设的各界人士，致以崇高的敬意和衷心的感谢！

在总结成绩的同时，我们也看到了当前存在的困难和问题。主要有：征地拆迁工作难度加大，用地供应和建设资金比较紧张；工业新上重大项目偏少，中小企业融资问题依然突出，改造提升传统产业任务艰巨；节能减排压力很大；县域经济发展不平衡，农村贫困地区发展途径不多；就业形势依然严峻，食品价格上涨较快，部分群众生活困难，民生问题有待进一步改善；交通枢纽地位亟待强化等等。所有这些，必须采取有效措施，认真加以解决。

2008年工作任务

党的十七大对全面建设小康社会提出了新的更高的要求。根据十七大精神，市委在市第十次党代会确立奋斗目标的基础上，提出柳州要在全区率先实现工业化、率先实现人均地区生产总值翻两番的新目标。我们必须适应形势发展的新变化，顺应全市各族人民过上更好生活的新期待，坚定信心，埋头苦干，为全面建设小康社会打下更加牢固的基础。

当前，宏观经济形势总体上对我市加快发展有利。从国际上看，经济全球化深入发展，国际和区域合作方兴未艾，国际产业转移和生产要素流动加速，世界经济仍将保持较快增长。从国内来看，我国正处于全面建设小康社会的关键时期，经济增长的内在动力持续增强，全国各地加快发展愿望迫切、热情高涨，国内经济多年高速增长所形成的巨大发展惯性，将有力推动国民经济继续保持高位运行态势。从全区来看，北部湾风生水起，各地发展百舸争流、千帆竞发，经济社会发展全面提速，广西进入了有史以来的最好发展时期。从我市来看，经过多年的持续快速发展，经济实力大幅提升，发展后劲明显增强，发展环境不断优化，柳州已具备实现跨越发展的基础和条件，全市人民充满热情和信心，正在朝着在全区率先实现工业化和城镇化的目标大步迈进。

各位代表，在看到经济发展有利条件的同时，我们也要对发展中的各种不利因素引起足够的重视。尤其是中央加大宏观调控力度，实施从紧的货币政策，加强和规范新开工项目管理，强化节能减排的刚性约束等，将会使我市项目核准、审批难度加大，土地瓶颈约束更加突出，建设资金筹措更加困难。面对复杂多变的形势，我们一定要常怀忧患之思，常有紧迫之感，灵活应对，因势利导，趋利避害，加快发展。

今年政府工作的总体要求是：深入贯彻落实党的十七大精神，坚持以科学发展观统领全局，紧紧围绕“工业强市，富民兴柳”总体发展思路，坚持“六个创新”，切实增强在新形势下创造性地加快发展的能力，努力做到工业发展与环境保护同步推进、国民经济与宜居城市同步发展、经济社会发展水平与人民生活水平同步提高（简称“三个同步”），加强经济建设、政治建设、文化建设和社会建设，推动经济社会又好又快发展，着力建设工业名城、历史名城、文化名城、旅游名城，打造“生态工业柳州，宜居创业城市”，为在全区率先实现工业化和城镇化、建设富裕文明和谐新柳州做出新的贡献。

今年要突出发展和民生两大主题，紧紧围绕投资项目建设促进年、文化社会建设促进年、宜居城市建设促进年，大力实施百亿投资工业强市工程、百亿投资城建美市工程、10亿投资农业稳市工程，50亿投资商贸旺市工程，20亿投资社会建设安市工程，10亿投资文化盛市工程，确保今年有更好、更快、更大的发展。

基于国际国内发展态势和我市2009年地区生产总值冲刺1000亿元、工业总产值超越2000亿元考虑，今年我市经济和社会发展的主要预期目标是：地区生产总值847亿元，增长14%；财政收入134亿元，增长15%；全社会固定资产投资400亿元，增长32%；工业总产值1680亿元，增长20%；单位生产总值能耗下降5%以上；主要污染物二氧化硫（SO2）、化学需氧量（COD）排放分别减少3.1%和3.9%；城镇居民人均可支配收入14150元，增长10%；农民人均纯收入3730元，增长10%；城镇登记失业率控制在4.5%以内；居民消费价格指数力争控制在全国平均涨幅之内；人口自然增长率控制在自治区下达指标以内。

为实现上述预期目标，着重抓好八个方面的工作：

一、实施百亿投资工业强市工程，加快有柳州特色的新型工业化进程

坚持工业主导战略，加快“柳州制造”向“柳州创造”的转变，坚定不移地走有柳州特色的新型工业化道路，逐步把柳州打造成为国际机械城和重要钢铁基地。切实加大工业投资力度，迅速扩大工业经济规模，确保今年工业总产值达1680亿元以上。

深入实施四大成长计划，开展“六个百亿”行动。集群发展三大支柱产业，改造提升传统优势产业，培育发展新兴产业，力争今年三大支柱产业产值突破1000亿元，五大传统产业产值突破100亿元，四大新兴产业产值超过30亿元。开展“六个百亿”行动，力争年内新增主营业务收入迈上300亿元、200亿元和100亿元新台阶的企业各一家，力争实现工业产值超百亿元的县、城区和工业园区各一个。继续开展“中小企业服务月”活动，进一步完善中小企业服务体系，创造更有利于中小企业发展的环境，鼓励和扶持中小企业加快发展，力争年内全市规模以上工业企业再增100户、产值过亿元企业达130户以上。鼓励和支持企业争创名牌，培植一批拥有自主知识产权和知名品牌的优势企业，每年争创1-2个中国名牌产品和驰名商标。

加强项目策划和建设，调整优化产业结构。今年全市工业投资要达到150亿元以上，其中工业技改投资达130亿元以上。推进上汽通用五菱、柳工、五菱集团等企业的一批重大技改项目，实现主机和配套产业同步发展；抓好柳钢结构调整项目，确保年内钢材产能达800万吨。加快培育发展以化工为代表的未来战略产业，着重推进柳化20万吨/年烧碱、20万吨/年聚氯乙烯工程等一批重大项目。抓住东部加工业转移机遇，大力发展服装加工、家具制造、包装印刷、食品加工等劳动密集型产业。

大力发展园区经济，推动工业产业集约发展。建立完善柳东新区投融资平台，加快柳东新区和各工业园区基础设施及产业项目建设，确保柳东新区和工业园区的产值与投资增速高于全市平均水平。大力发展“飞地经济”，统筹实施园区发展规划。在柳东新区筹建县区工业集中区和农产品加工园区，尽量满足各县区发展工业需要。结合国家汽车及零部件生产基地、出口基地建设，规划并启动柳工工业园、上汽通用五菱工业园等项目，促进汽车及零部件、机械工业进入园区集约发展，打造特色产业基地。

加强自主创新，推动企业技术进步。组织实施第四轮创新计划，开展创新型企业试点。完善公共技术服务平台，推动企业信息化建设，争取建立一个国家级工程技术研究中心。认真抓好国家茧丝绸产品质检中心及铟、汽车零部件、预应力等自治区级产品质检中心的建设。加大对重点领域、重大专项或关键环节的科技投入。加大专利资助及奖励力度，激励企业积极参与国家标准和行业标准的制定。

加强节能减排工作，推动工业发展与环境保护友好共进。建立健全节能减排工作机制，落实节能减排任务。积极推广先进适用环保节能技术，深入实施能源替代计划，着重抓好重点企业的节能降耗，确保实现年度能耗下降与主要污染物减排目标。大力发展循环经济，持续推进柳钢、柳化等企业的循环经济示范项目，建设柳北工业区循环经济网络，力争成为国家第二批循环经济试点城市。

加大扶持力度，优化企业发展环境。进一步完善成长计划配套政策和激励机制。市财政每年安排扶持工业企业发展资金增长的幅度不低于财政收入增长的幅度。推进产业投融资平台建设，建立健全风险投资机制，逐步扩大工业投资规模；将国有资产收益和工业其他收益进行产业投资，积极探索国有资本产业投资的新形式，做到退而有序、进而有效，扶持有潜力的企业或项目迅速成长。鼓励和支持各类担保公司快速发展，逐步建立有柳州特色的融资担保服务体系。推进市区企业搬迁改造，盘活土地资源和国有资产。支持企业上市融资，力争年内实现1-2家企业上市。加强人才队伍建设，加快引智引才步伐，建设“人才小高地”，打造优秀的企业家队伍，为经济社会发展提供强有力的人才支撑。

二、实施10亿投资农业稳市工程，进一步强化农业的基础地位

坚持用工业的理念谋划农业发展，建立以工促农、以城带乡长效机制，扎实推进社会主义新农村建设，推动农村发展，促进农业增效，确保农民增收。今年农林牧渔业总产值要达到145亿元，增长10%以上。

加快发展现代农业，推进农业产业化。稳定粮食生产，大力发展甘蔗、桑蚕、水果、蔬菜、茶叶、茶油、中药材等优势产业。积极推进品种改良，探索发展多种形式的适度规模经营，着力扶持一批500亩、1000亩的甘蔗种植大户，积极扶持桑蚕茧成为产业化经营的龙头产业。深入实施龙头企业成长计划，引导龙头企业增创品牌和扩大带动效应。围绕竹浆造纸业发展，成立造林公司，打造300万亩的竹林基地。加强对农业专业合作组织的培育和引导，增强农民参与市场竞争的能力，提高农产品市场化程度。继续实施“千村两机”工程，加快农业机械化进程。

以养殖业为切入点，努力实现农业发展的新突破。切实加大养殖业扶持力度，大力发展生猪、肉牛和水产养殖，着重扶持一批养殖大户，引进和培育一批养殖龙头企业，打造农业发展新支柱。市财政在保持原来投资的基础上，将每年增加的农业生产性投资的50%用于扶持养殖业发展，并每年再追加养殖业发展资金300万元。今年要确保养殖业产值达60亿元，养殖业占农林牧渔业总产值比重达42%以上，力争2010年达45%以上。

大力发展县域工业。鼓励和支持各县围绕城市工业配套、产业转移及填补产业空白，大力发展各具特色的县域工业，打造市县一体的产业分工协作体系。鼓励各县加大投入，合理规划和集约利用土地，大力发展园区经济，做大县域工业。

加大投入力度，加强农村基础建设。大力推进以农田水利和农村道路交通为重点的基础设施建设。今年要建设通村、通屯道路400公里，建成通乡油路3条134公里，实现乡乡通油路。继续抓好农村饮水安全、沼气池、卫生改厕、水库除险加固、茅草房改造等项目建设。着力改善城郊农村基础设施落后状况。全部解决柳州电网范围内的无电户用电问题。持续推进县城和一批重点城镇建设，不断提高城镇的承载能力。以项目为载体，扎实推进社会主义新农村建设和生态富民小康示范样板村建设，年内新增32个县区新农村建设示范点。

开展村寨防火改造工程大会战。加大市、县财政投入力度，发动村民投工投劳，开展水改、电改、灶改、寨改和房改的“4+1”村寨防火改造工程大会战，争取在三年内消除村寨重大火灾隐患。

三、实施50亿投资商贸旺市工程，打造区域性商贸物流中心

坚持依托并服务于一产、二产，密切三次产业联结，推动第三产业快速发展，繁荣市场，聚集人气，振兴桂中商埠，努力打造区域性商贸物流中心。今年全市社会消费品零售总额要达到320亿元，增长17%以上，努力提高我市三产在全区的比重。

大力发展商贸物流业。实施《“十一五”商贸业发展规划》，出台《关于加快服务业发展的决定》和相关配套文件，加大对商贸服务业发展的扶持力度。一是推进市场培育与建设。着力打造一个年交易额超100亿元、两个超50亿元的大市场，建设一批10-50亿元的专业批发市场。继续改造提升柳邕农副产品批发市场和一批粗放式的农贸市场，加快东环、广雅市场建设，进一步完善河东、河西及旧机场片区的商业服务功能。推进五星、工贸整合，打造10亿元的商业零售龙头企业。二是依托工业和交通优势，建立区域性商贸物流体系。加大物流基础设施投入，加快桂中海迅、生产资料、阳和口岸等物流基地建设，做大做强一批现代物流企业。围绕区域性一级批发市场建设，大力发展前店后厂的产业模式，促进小商品生产与贸易发展。三是加强农村市场网络建设，深入实施“万村千乡工程”和“信福工程②”；实施“双百市场工程③”，促进农产品流通。

加大市场调控和消费引导力度。深入实施食品安全工程，积极开展“便民早餐④”工程试点。落实国务院有关扶持生猪生产政策，继续采取有效措施，稳定肉食品市场供应。积极挖掘地方特色菜系，打造柳州本土餐饮品牌；筹划建设特色餐饮一条街，推动餐饮业发展。引导和规范房地产业健康发展，鼓励和吸引周边市县居民到我市购房置业。鼓励柳州人买柳州车，柳州人在柳州买车，培育柳州汽车市场。

努力构筑大旅游发展格局。加强景观策划，有序推进“百里柳江”、“百里风情”旅游重点项目开发。发展城市水上旅游，投放豪华游船和水上的士，开发水上娱乐项目。深入挖掘少数民族风情和非物质文化遗产，加快三江程阳八寨、贝江、元宝山等项目的开发建设，打造民族风情旅游线路精品。大力发展环都市农业生态观光休闲旅游。加强区域合作，主动融入大桂林旅游圈，努力拓展海内外旅游市场。

加快发展新型服务业。积极推动各类生产性服务业、中介服务业和会展业发展，大力发展科技、教育、文化、医疗等现代服务业，不断壮大社区服务、社会化养老和物业管理等新兴服务业。加快金融业发展，打造良好金融生态环境，建立政银企良性互动合作机制，增强金融业支持经济社会建设的力度。

四、实施百亿投资城建美市工程，强力推进宜居城市建设

坚持高水平规划、高强度投入、高标准建设、高质量管理，着力完善城市功能，提升城市品质，塑造城市新形象，彰显山清水秀地干净的城市新风貌，优化城市人居和创业环境。

加强规划和用地管理。贯彻落实《城乡规划法》，进一步提高规划编制水平，完成城市总体规划和新一轮土地利用总体规划修编，加强城镇控制性详规的编制。严格依法执行规划，维护规划的严肃性和权威性。千方百计解决土地瓶颈问题，充分利用我市农场山林土地，建立节约集约利用土地机制，推进土地战略储备，努力保障建设用地供应。

完善城市功能，加强城市基础设施建设。一是极力拓展对外交通，巩固提升柳州的交通枢纽地位。成立柳肇铁路、柳粤高速、柳江二级航道和柳州航线发展四个推进委员会，积极争取国家、自治区和各方面的支持，统筹策划和推进铁路、公路、航道建设和航线发展。积极配合做好黔桂、湘桂铁路扩能改造。开展柳州—三江高速公路前期工作。尽快启动白莲机场扩建工程。疏浚柳江航道，提高通航能力。二是继续完善城市路网配套。启动柳州城市快速环道建设，打通环形交通；开工建设广雅大桥等三座大桥，把柳州打造成为桥梁博物馆；改善内环路及中心主骨架道路交通，竣工柳长路南段、雅儒路北段、北雀路、白沙路等道路，改造屏山大道、荣军路等道路。改造重要交通节点，建设鱼峰路、西江路等路口下穿通道，竣工龙城路、续建五一路地下工程。三是加快柳东新区基础设施建设。完成静兰桥改建，加快火炬大道建设，拓展新区中心路网，不断完善新区排水工程。

提升城市品质，打造山水城市新风貌。一是着力打造城市景观新亮点。新开工四条沿江景观道路，继续打造世界最宽的人工瀑布群，启动柳州风情港、沙角观瀑广场和人工沙滩浴场建设；实施萝卜洲抢救性保护和生态恢复工程；策划在人民医院现址建设地标性建筑。二是加大旧城改造力度。坚持以城区为主推动城中村改造，以项目建设带动老城区改造，启动改造静兰、窑埠、红光、白沙、黄村等片区城中村，继续实施穿衣戴帽工程，改造一批小街小巷。推进排水设施建设，解决城市低洼地带内涝问题。三是加强园林绿化工作，争创国家园林城市。推进驾鹤小桃源、河西花卉公园、蜡烛山公园的二期工程和马鹿山奇石博览园建设，开工建设北雀广场、土伏山公园，启动柳州植物园、大型花卉公园前期工作。加强城市绿化和山体景观保护，开展“百万人种百万棵树”活动。扩大柳江沿岸柳树种植，打造“百里柳江·十里柳堤”。四是加强大气、水体、噪声、扬尘和固废污染的综合治理。完成第一次全国污染源普查。加强重点污染源治理。实施柳钢烟气脱硫工程，关闭白莲淀粉厂，推动柳锌二基地搬迁，推进造纸、制糖行业的污染治理。加强环保基础设施建设。竣工竹鹅溪综合治理南支渠工程，确保白沙、新兴工业园等污水处理厂投入运行，建成阳和和柳江、鹿寨、柳城三县的污水处理厂；加强柳江上游水源林保护，启动融安、融水、三江三县污水处理厂前期工作，确保2009年建成。力争市区大气质量达到国家二级标准，柳江水质保持国家地表水Ⅲ类标准。

塑造城市新形象，提高市民素质，加强城市管理。深化“城乡清洁工程”和“五项整治”，继续开展“南珠杯”竞赛活动，加强农贸市场、城中村、城郊和城铁结合部、无人管理住宅小区等薄弱环节的专项整治；继续抓好公厕、垃圾中转站、医疗废物处置等环卫设施建设。推进示范街、示范市场、示范小区建设。全面推进城市管理综合执法，加快“数字城管”建设，健全长效管理机制。加强舆论引导，强化市民意识，提高市民素质。加强城市形象策划，扩大城市宣传，塑造“生态工业柳州，宜居创业城市”新形象。

五、主动参与多区域合作，加快推进改革开放

进一步深化各项改革，优化投资环境，积极营造亲商、安商、富商的良好氛围，努力提高外向型经济水平，增强经济发展的动力与活力。今年引进市外境内资金要达到200亿元，实际利用外资突破1亿美元，出口总值达到8.7亿美元，增长30%。

加大招商引资力度。围绕产业发展、城市建设、园区开发等重点，精心策划项目，充分利用商会、信息网络、对外交流三大平台，全面开展招商引资。改进招商机制和招商模式，做到“三结合三为主”：实行统一招商和分口招商相结合，以部门、县区、园区分口招商为主；实行大型招商与小型招商相结合，以小分队招商和点对点招商为主，直接进行项目对接和落实；坚持“走出去”和“请进来”相结合，以“请进来”为主，增强招商的实效性。制定并落实奖励政策，动员、鼓励社会力量参与招商。成立招商促进服务中心、投资投诉服务中心，建立项目审批“绿色通道”，不断优化投资环境。抓好承接东部产业转移工作，在东莞设立外联常设机构，促成东部产业项目向我市转移，争取成为国家承接产业转移基地。

加快外经外贸发展。优化出口产品结构，巩固传统大宗商品出口，努力扩大机电产品及有色金属新材料、高新技术产品出口。积极开拓海外市场，推进出口市场多元化，力争工程机械、汽车、化工、农产品几大类产品进入东盟市场有新的突破。加大“走出去”力度，坚持以项目合作带动产品、技术、劳务出口；积极筹划“北部湾柳州工业园”建设，组织和引导企业入园发展。加强口岸建设，进一步优化通关环境。

深化体制改革。进一步完善国有资产管理体制，积极推进国有资本经营预算改革，建立国有资产收益专户，改进预算编制和管理方式。不断革除体制障碍，着力改善民营企业、非公经济的发展环境。加强政府主导、市场运作、社会参与的多元化投融资平台建设，增强国有投资公司投融资功能，推进公共事业资产所有权和使用权分离，把财政投融资转变为国有投资公司市场化运作，引导和带动社会参与公共事业投资。深化城市管理体制改革，理顺城区管理体制，加强社区建设和管理。加强侨务工作，抓好华侨农场管理体制改革。继续推进农村综合改革试点。加快推进事业单位分类改革，全面推行事业单位聘用制度。深化文化体制改革，确保专业文艺团体和演出公司体制改革取得成效。

六、实施20亿投资社会建设安市工程，加强以改善民生为重点的社会建设

加大公共财政支出力度，加快推进以改善民生为重点的社会建设，着力解决关系人民群众切身利益的困难和问题，努力为人民群众办实事，让人民群众共享改革发展成果，促进社会和谐稳定。

优先发展教育。巩固提高“两基”成果，整合义务教育资源，大力推进义务教育均衡发展。进一步提高高中阶段普及率。全面推进素质教育，深化教育改革，加强教师队伍建设，不断提高教育质量。扶持发展特殊教育。继续实施教育扶贫行动，全面贯彻落实农村义务教育保障新机制及“两免一补”等政策，着力完善家庭经济困难学生资助政策体系。努力缓解边远山区学校教师住房困难。全面实施职教攻坚。积极引进民间和社会资金参与职业院校建设，推进职业教育投入多元化；加快推进职教区建设，争取首期工程年内开工。加强校园文化建设，推动学校内涵式发展，凸显办学特色。

实施扩大就业的发展战略。坚持实施积极的就业政策，贯彻落实《就业促进法》，开展全民创业活动，鼓励支持自主创业、自谋职业。建立和完善就业援助长效机制，加大对就业困难群体的帮扶力度，着力帮助下岗失业人员和零就业家庭解决就业问题。推进统筹城乡就业，健全覆盖城乡劳动者的就业管理服务体系，加大农村劳动力技能培训和转移就业工作力度，加强对大中专毕业生、退伍转业军人、失地农民的就业指导和服务。贯彻实施《劳动合同法》，加大劳动保障监察执法力度，不断完善农民工工资保障金制度，依法维护劳动者权益。争取城镇新增就业人数4.5万人，实现农村富余劳动力转移就业4万人。

完善覆盖城乡的社会保障体系。进一步规范城乡最低生活保障制度，巩固应保尽保，推进分类施保。继续调整提高企业退休人员基本养老金。加强社会保险扩面征缴，力争年内实现五险合一统一征缴、一票征收。继续推进农民工参加工伤保险的“平安计划”。制定出台被征地农民社会保障制度等相关政策。建立健全城乡社会救助体系。完善临时救助制度和城乡医疗救助制度。健全农村五保和城镇“三无”对象⑤集中供养长效机制，加强五保村、乡镇敬老院和社会福利院建设。积极发展社会福利事业。

加强公共卫生建设。积极推进医疗卫生体制改革。进一步优化城市卫生服务结构，开展社区基本医疗药品零差价试点。突出抓好医疗服务质量。加快市人民医院和市疾病预防控制中心搬迁步伐，筹建城区卫生监督所，切实抓好六县和城郊乡镇卫生院项目建设，推进企业家保健中心建设。继续推进城镇居民基本医疗保险试点，全面推行新型农村合作医疗，提高财政补助水平。继续抓好农村初级卫生保健工作，确保80%以上的县（区）初保达标。启动第四周期城市卫生支援农村卫生工作，继续开展“万名医师支援农村卫生”活动。强化疾病预防控制，重点抓好重大传染病的防控工作。

切实解决关系群众切身利益的困难和问题。采取积极有效措施，想方设法抑制猪肉等副食品价格上涨势头，努力缓解物价上涨对贫困家庭生活的影响。完善廉租住房制度，适当扩大廉租范围和经济适用房建设规模，全年新开工建设廉租房和经济适用房38.3万平方米，努力解决城市中低收入家庭的住房问题。继续实施关爱残疾人“阳光计划”，积极开展关爱残疾人的活动和救助工作。适应老龄化社会进程加快的新形势，大力发展托老、养老和老年福利事业，不断完善老年人学习、健身、休闲、娱乐设施和场所，给予老年人更多的关爱。精神文化生活。深入推进扶贫开发，继续实施融水、三江易地扶贫搬迁工程，全面推进第二批85个贫困村“整村推进”扶贫开发，深入开展大中型企业与乡镇、村屯结对子帮扶活动，带动贫困乡村发展。

维护社会安定团结。强化社会治安综合治理，深入实施社区和农村警务战略，进一步健全和完善社会治安防控体系，继续推进打击“两抢一盗”、打黑除恶、命案侦破等专项行动。加强以抓基层、打基础、苦练基本功为主要内容的“三基”工程建设，推进科技强警，全面建成“天网”工程⑥。加强信访工作，开展干部下访专项活动，加大“三大纠纷”调处力度。高度重视安全生产，树立安全发展理念，开展“隐患治理年”活动。健全灾害应急救助机制，努力提高灾害救助水平。

七、实施10亿投资文化盛市工程，丰富群众精神文化生活

坚持社会主义先进文化前进方向，推进文化创新，大力发展具有时代特征、地方和民族特色的文化事业和文化产业，推动文化大发展大繁荣，让人民群众共享文化发展成果。

大力发展文化体育事业。繁荣文艺创作，精心打造精品剧目舞剧《刘三姐》和动画教育电视连续剧《心灵橱窗》，推进《白莲》、《八桂大歌》等艺术精品商业化巡演。加大对历史文化、民族民间文化和非物质文化遗产的保护和开发力度，搞好第三次文物普查。不断完善城乡基层文化服务体系，加快市工人文化宫和群众艺术馆、各城区图书馆、文化馆、乡镇文化站以及市级文化信息资源共享中心、文化信息网的建设。精心筹办第五届柳州国际奇石节和“2008欢乐柳州·国际小丑狂欢节”。加强广播电视和新闻出版工作，加大“扫黄、打非”力度，继续实施“村村通”广播电视工程和“农家书屋”建设工程。抓好新一轮市志编纂工作。广泛开展全民健身运动，举办高水平的国际体育赛事。加强公共体育设施建设，争取开工建设市体育馆、游泳馆、八一路体育休闲广场等体育项目。

大力发展具有柳州特色的文化产业。高起点编制全市文化产业发展总体规划，着力打造“一园、一片、一江、一线、一基地”文化产业新格局。“一园”即通过打造国际性主题欢乐文化公园、文化产业园、动漫产业园、印刷产业园、白莲洞古人类生态园等若干个专业园区，连片构成大文化产业园。“一片”即以河北半岛原有文化娱乐设施为阵地，不断提炼产业元素，提升产业水平，形成河北半岛文化产业聚集片。“一江”即统筹推进柳江沿岸文化产业建设，着重打造柳州风情港、刘三姐大剧院、水上观光游览等沿江文化项目，建设“百里柳江·百里舞台”。“一线”即整合开发三江侗族生态文化、融水苗族风情文化、融安古镇民间文化、柳城壮族村戏文化资源，打造独具特色的柳北四县民族风情生态文化旅游线。“一基地”即整合市内高等院校、高中级职业院校、艺术学校等优势资源，打造文化产业、创意产业、动漫产业、演艺产业人才培训基地。推进文化产业资源整合，推动文化产业项目建设。

进一步加强精神文明建设。深入学习和贯彻落实党的十七大精神，切实把社会主义核心价值体系融入精神文明建设全过程，努力提高城市文明程度和市民文明素质。繁荣和发展哲学社会科学。深入贯彻公民道德建设实施纲要，认真实施妇女儿童发展纲要，加强和改进未成年人思想道德建设。积极开展中小学生科普教育，加强基层科普场所建设，提高未成年人的科学素养。广泛开展文明村镇、文明社区、文明行业、文明单位和“迎奥运、讲文明、树新风”等精神文明创建活动；加强诚信建设，争创自治区和全国文明城市。继续抓好国防教育、国防动员和双拥共建工作，巩固军政、军民团结。加大对少数民族和民族地区经济社会发展的扶持力度，促进民族团结，实现共同繁荣。

八、坚持依法行政、从严治政，建设阳光、务实、高效的服务型政府

努力提高行政水平。正确履行政府职能，加强公共财政建设，强化公共管理和公共服务，不断加强和改善经济社会发展的薄弱环节。完善科学民主决策机制，坚持专家咨询制度、社会公示和听证制度，进一步提高行政决策水平。坚持从严治政，各级各部门对市委、市政府的决定要坚决做到令行禁止，步调一致；坚决杜绝部门利益，严肃查处乱作为、不作为行为，做到言必信、行必果，不断提高政府的执行力和公信力。

切实提高行政效能。深化行政审批制度改革，简化审批环节，规范审批程序，推进审管分离和并联审批，大力推行网上审批，不断完善市县（区）政务服务中心功能和电子监察系统功能。深入开展行政效能建设，全面落实“三项制度”和否定报备制度，切实加大行政效能监察和投诉查处工作力度，深入开展效能质询活动，促进机关作风的根本好转。实行“阳光政务”，推进政务公开，加强“12345”政府热线、绿色通道、市长信箱、政府门户网站等互动平台建设，广开言路，集中民智，主动接受社会公众监督。

加强民主法治建设。各级政府要自觉接受同级人大及其常委会的监督，加强与政协的联系，认真听取民主党派、工商联、无党派人士和各人民团体的意见。落实《全面推进依法行政实施纲要》，全面推进依法治市。切实加强以农村、社区为重点的基层民主法制建设，健全基层群众自治机制，扩大基层群众自治范围，完善民主管理制度。积极推进厂务公开，完善村务公开。深入开展“五五”普法，形成自觉学法守法用法的社会氛围。

加强党风廉政建设。建立健全惩治和预防腐败体系，落实党风廉政建设责任制，切实抓好领导干部和国有企业领导人员廉洁自律工作。加大专项治理力度，坚决纠正损害群众利益的不正之风。抓住关键环节，深入整治商业贿赂并建立起长效机制。完善资金管理和使用制度，强化对政府性、公共性资金的使用管理和审计监督。

各位代表，回顾过去的一年，我们为取得的成绩欢欣鼓舞；展望新的一年，我们豪情满怀，对建设柳州新的伟大事业充满必胜的信心，我们期待柳州明天会更好。让我们乘着十七大的东风，以邓小平理论和“三个代表”重要思想为指导，深入贯彻落实科学发展观，在市委的领导下，振奋精神，开拓进取，扎实工作，为建设富裕文明和谐新柳州做出新的贡献，以优异的成绩向自治区成立五十周年大庆献礼！
